# Outcomes of surgical treatment of diverticular abscesses after failure of antibiotic therapy

**DOI:** 10.1007/s13304-023-01509-4

**Published:** 2023-04-24

**Authors:** Alberto Arezzo, Antonella Nicotera, Luca Domenico Bonomo, Francesco Olandese, Simona Veglia, Alice Ferguglia, Giuseppe Pentassuglia, Giuseppe Mingrone, Mario Morino

**Affiliations:** 1 Department of Surgical Sciences, University of Turin, Corso Dogliotti 14, 10126 Turin, Italy; 2 Department of Diagnostic Imaging and Radiotherapy, AOU Città della Salute e della Scienza di Torino-University of Turin, Turin, Italy

**Keywords:** Acute diverticulitis, Diverticular abscess, Emergency surgery, Percutaneous drainage, Antibiotic therapy, Observational study

## Abstract

Management of diverticular abscess (DA) is still controversial. Antibiotic therapy is indicated in abscesses ≤ 4 cm, while percutaneous drainage/surgery in abscesses > 4 cm. The study aims to assess the role of antibiotics and surgical treatments in patients affected by DA. We retrospectively analyzed 100 consecutive patients with DA between 2013 and 2020, with a minimum follow-up of 12 months. They were divided into two groups depending on abscess size ≤ or > 4 cm (group 1 and group 2, respectively). All patients were initially treated with intravenous antibiotics. Surgery was considered in patients with generalized peritonitis at admission or after the failure of antibiotic therapy. The primary endpoint was to compare recurrence rates for antibiotics and surgery. The secondary endpoint was to assess the failure rate of each antibiotic regimen resulting in surgery. In group 1, 31 (72.1%) patients were conservatively treated and 12 (27.9%) underwent surgery. In group 2, percentages were respectively 50.9% (29 patients) and 49.1% (28 patients). We observed 4 recurrences in group 1 and 6 in group 2. Recurrence required surgery in 3 patients/group. We administered amoxicillin-clavulanic acid to 74 patients, piperacillin-tazobactam to 14 patients and ciprofloxacin + metronidazole to 12 patients. All patients referred to surgery had been previously treated with amoxicillin-Powered by Editorial Manager^®^ and ProduXion Manager^®^ from Aries Systems Corporation clavulanic acid. No percutaneous drainage was performed in a hundred consecutive patients. Surgical treatment was associated with a lower risk of recurrence in patients with abscess > 4 cm, compared to antibiotics. Amoxicillin-clavulanic acid was associated with a higher therapeutic failure rate than piperacillin-tazobactam/ciprofloxacin + metronidazole.

## Introduction

Diverticular abscess (DA) is the most frequent complication of acute colonic diverticulitis (AD). It is usually diagnosed in approximately 15–20% [1–4] of AD cases, mostly using contrast-enhanced-computed-tomography scan (CT-scan) [5]. DAs are classified according to location and size and the most widely used classifications for staging are the best-known Hinchey’s [6] and the most recent one from the World Society of Emergency Surgery (WSES) [7]. According to Hinchey’s modified classification, DA can be confined in pericolic fat, smaller than 5 cm (stage Ib) or distant intra-abdominal/retroperitoneal at least 5 cm in size (stage II). Differently, according to WSES, the Ib stage indicates the presence of an abscess ≤ 4 cm, while the IIa stage indicates an abscess > 4 cm.

While for the uncomplicated forms of AD conservative treatment is established, and the treatment of the severe cases is surely surgical, the management of intra-abdominal DAs is still controversial. Conservative treatment is currently suggested in small abscesses (size ≤ 4 cm), while percutaneous drainage (PD) or surgery are suggested in larger abscesses [8–10]. Here the role of surgery is debated, being necessary to distinguish between elective and emergency surgery. Elective surgery is considered for patients after the resolution of acute episodes and consists of colic resection and primary anastomosis (PA). The indication is based on the quality of life, the number of acute episodes and the wellness intervals, the immunity status and the acceptance of possible complications [7]. On the contrary, the indication for emergency surgery is accepted in case of severe complications of AD, such as extensive abdominal collections, or entero-visceral fistulas, as well as after the failure of conservative treatment, such as antibiotics or percutaneous drainage. Emergency surgery is burdened by a high mortality rate, often due to co-morbidities or sepsis, rather than the technique itself.

The study aimed to assess the role of antibiotics and surgery in patients affected by DA and the effectiveness of different antibiotic regimens.

## Methods

We retrospectively analysed a prospective database, including all consecutive patients with contrast-enhanced CT scan diagnosis of acute Hinchey Ib–II and WSES Ib–IIa diverticulitis of the left colon (from the splenic flexure to the rectum), admitted to our hospital between January 1st, 2013, and December 31st, 2020. Exclusion criteria were age < 18 years, co-presence of other acute diseases, and use of oral anticoagulant therapy. We considered a minimum follow-up period of 12 months after discharge from the hospital. The study was conducted in good clinical practice according to the Helsinki Declaration of 1975 and subsequent modifications. The information has been processed after checking the presence of informed consent to the processing of personal data for purpose of scientific research.

After CT scan diagnosis and staging, all patients were initially referred to conservative treatment. In our series there were no cases of generalized peritonitis; otherwise, such patients would have been referred for upfront surgery.

Conservative treatment consisted of intravenous (i.v.) administration of amoxicillin + clavulanic acid (2.2 g dose every 8 h per 6 days), or ciprofloxacin (400 mg dose every 12 h per 6 days) + metronidazole (500 mg dose every 8 h per 6 days), or piperacillin + tazobactam (4.5 g dose every 8 h per 5 days). The choice of the antibiotic regimen was based on the preference of the medical doctor responsible for the patient’s management. The antibiotic therapy was defined as successful when we observed a resolution of the acute infection, the regression of symptoms and the normalization of inflammatory indices. We considered the antibiotic therapy to fail when observing persistent symptoms, increased white blood cell (WBC) count and C-reactive protein (CRP) dosage after 48 h from the start of treatment. Failure of antibiotic therapy led to surgical treatment. This consisted of colic resection with PA with or without a protective stoma or Hartmann’s procedure (HP) according to the operator’s choice.

Per each patient, the following characteristics were considered: age, gender, body mass index (BMI), Charleston Comorbidity Index (CCI), smoking, family history of diverticulitis disease (DD), comorbidities, AD location, abscess location, abscess size (mm), white blood cells (WBC) count at admission, history of AD, previous surgery for AD.

### Endpoints

The primary endpoint was to compare recurrence rates for conservative (antibiotics) and surgical therapy in patients affected by DA. The secondary endpoint was to assess the failure rate of each antibiotic regimen resulting in surgery.

### Subgroups

**Fig. 1:**
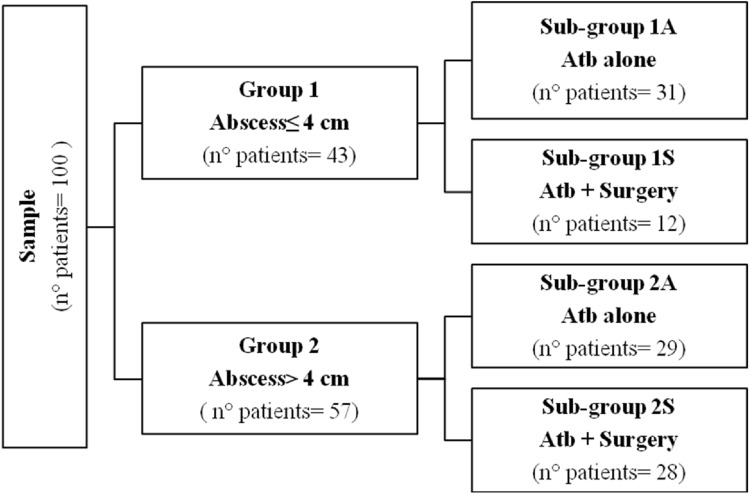
Trial design

The series was divided into two groups according to WSES classification: patients with DA size ≤ 4 cm (group 1) and patients with DA size > 4 cm (group 2). Each group was further divided based on treatment: antibiotic therapy (sub-group 1A/2A) vs antibiotic + surgery (sub-group 1S/2S) (Fig. 1).

### Statistical analysis

Data were analysed using descriptive and inferential statistics. Categorical variables were expressed as absolute/relative frequencies, while the continuous ones as mean (standard deviation) or median (range). Statistical analysis was conducted with a chi-square test for categorical variables. For continuous variables, Student’s *t* test and Mann–Whitney *U* test were used, as appropriate. The significance level was set at 5% for all variables. All statistics were performed with SPSS 22.0 (SPSS Inc., Chicago, IL).

## Results

A hundred consecutive patients were included in the study. The mean age of the whole series was 61 years (± 15), with a little prevalence of women (51%). Median CCI at diagnosis was 2 (0–8). All patients’ characteristics are reported in Table 1. The most common comorbidity was cardiovascular disease (22%). In the majority of cases, AD was located in the sigmoid colon (94%), while the abscess was in most of the cases pericolic/mesocolic (68%).

**Table 1:** Patients’ main characteristics

	Patients n = 100
Age (mean, SD)	61 (± 15)
Males, *n* (%)	49 (49)
Body Mass Index (mean, SD)	27 (± 5)
CCI (median, range)	2 (0–8)
Smoking, *n* (%)	16 (16)
Family history of DD, *n* (%)	0
Comorbidities, *n* (%)	
Cardiovascular	22 (22)
Diabetes mellitus	9 (9)
Pulmonary	5 (5)
Immunodeficiency	2 (2)
AD location, *n* (%)	
Left colon	5 (5)
Sigmoid colon	94 (94)
Rectum	1 (1)
Abscess location, *n* (%)	
Intramural	2 (2)
Pericolic/mesocolic	68 (68)
Retroperitoneal	0
Pelvic	30 (30)
Abscess ≤ 4 cm, *n* (%)	43 (43)
WBC at admission, 10^3^ uL (mean, SD)	14 (± 5)
Past history of AD, *n* (%)	18 (18)
Previous surgery for DD, *n* (%)	1 (1)

*SD* standard deviation, *CCI* Charlson Comorbidity Index, *DD* diverticular disease, *AD* acute diverticulitis, *WBC* white blood cells

Forty-three patients were included in group 1 (abscess ≤ 4 cm), while 57 were assigned to group 2 (abscess > 4 cm). In group 1, 31 patients (72.1%) were successfully treated with antibiotic therapy and 12 (27.9%) required surgery. In group 2, 29 (50.9%) cases received only antibiotic therapy and 28 (49.1%) underwent surgery after medical therapy failure during the same hospitalization.

Table 2 shows the comparison of the subgroups (antibiotic therapy A vs antibiotic + surgery S in each main group) for patients’ and disease characteristics. In group 1, the subgroups resulted homogeneous for all the variables considered except for “history of AD”, which was significantly more frequent both overall (n = 2 vs n = 16, *p* < 0.001) and in subgroup S (n = 1 vs n = 11, *p* < 0.001). All the 12 cases who underwent surgery were subjected to PA. In a single case, a protective loop ileostomy was performed. In group 2, subgroups A and S presented a significant difference in terms of median abscess size (55 mm vs 65 mm, *p* = 0.003). Groups 2A and 2S resulted homogeneous for all the other variables analyzed. Among the patients of subgroup 2S, 26 (92.9%) underwent PA (3 protected by loop ileostomy), while in the other 2 (7.1%) an HP was performed. Patients undergoing HP were > 70 years of age, both with a past history of AD, with abscess size > 50 mm and ASA IV. Also, at first observation they had body temperature > 38 °C and WBC > 15 × 10^9^ cells. In the 1S subgroup laparotomy was performed in 2 (16.7%) patients, laparoscopy in 7 (58.3%) patients and laparoscopy converted into an open approach in 3 (25%) patients. In the subgroup 2S percentages were respectively 39.3% (11 patients), 42.9% (12 patients) and 17.8% (5 patients). Characteristics of patients who underwent surgery in the two groups are reported in Tables 3, 4.

**Table 2:** Comparison of patients’ characteristics in the sub-groups (antibiotic therapy/surgery)

	Group 1 Abscess ≤ 4 cm (n = 43)			Group 2 Abscess > 4 cm (n = 57)		
	Atb (n = 31)	Atb + surgery (n = 12)	*p*	Atb (n = 29)	Atb + surgery (n = 28)	*p*
Age (mean, SD)	56 (± 17)	55 (± 8)	0.917	61 (± 16)	67 (± 13)	0.122
Males, *n* (%)	13 (41.9)	9 (75)	0.088	15 (51.7)	12 (42.9)	0.599
Body Mass Index (mean, SD)	28 (± 5)	26 (± 5)	0.385	27 (± 4)	27 (± 7)	0.993
CCI (median, range)	2 (0–8)	1 (0–3)	0.355	2 (0–8)	3 (0–6)	0.422
Smoking, *n* (%)	5 (16.1)	3 (25)	0.665	5 (17.2)	3 (10.7)	0.706
Family history of DD, *n* (%)	–	–		–	–	
Comorbidities, *n* (%)						
Cardiovascular	5 (16.1)	2 (16.7)	1.000	7 (24.1)	8 (28.6)	0.770
Diabetes mellitus	3 (9.7)	–	0.548	4 (13.8)	2 (7.1)	0.670
Pulmonary	3 (9.7)	–	0.548	1 (3.5)	1 (3.6)	1.000
Immunodeficiency	2 (6.5)	–	1.000	–	–	
AD location, *n* (%)						
Left colon	2 (6.5)	–	1.000	3 (10.3)	–	0.237
Sigmoid colon	29 (93.5)	12 (100)	1.000	25 (86.2)	28 (100)	0.112
Rectum	–	–		1 (3.5)	–	1.000
Abscess location, *n* (%)						
Intramural	1 (3.2)	1 (8.3)	0.485	–	–	
Pericolic/mesocolic	24 (77.4)	8 (66.7)	0.467	19 (65.5)	17 (60.7)	0.787
Retroperitoneal	–	–		–	–	
Pelvic	6 (19.4)	3 (25)	0.692	10 (34.5)	11 (39.3)	0.787
Abscess size in mm (median, range)	30 (10–40)	25 (10–40)	0.277	55 (43–80)	65 (50–220)	**0.003**
WBC at admission, 10^3^ uL (mean SD)	13 (± 4)	13 (± 4)	0.971	16 (± 7)	15 (± 6)	0.875
Past history of AD, *n* (%)	1 (3.2)	11 (91.7)	** < 0.001**	1 (3.5)	5 (17.9)	0.102
Previous surgery for DD, *n* (%)	1 (3.2)	–	1.000	–	–	

Statistically significant *p*-values are displayed in bold

*Atb* antibiotic therapy, *SD* standard deviation, *CCI* Charlson Comorbidity Index, *DD* diverticular disease, *AD* acute diverticulitis, *WBC* white blood cells

**Table 3:** Characteristics of patients with abscess ≤ 4 cm who underwent surgical treatment

	Type of surgery	Surgical approach	Clavien–Dindo ≥ 3	Readmission	Resurgery	Stoma reversal
HP	0	–	0	0	0	–
PA	11	2 lpt, 7 vls, 2 conv	0	0	0	–
PA + ileo	1	1 conv	0	0	0	1
Total	12	12	0	0	0	1

*HP* Hartmann’s procedure, *PA* colic resection and primary anastomosis, *PA* + *ileo* primary anastomosis with ileostomy, *lpt* laparotomy, *vls* laparoscopy, *conv* initial laparoscopy converted into open approach

**Table 4:** Characteristics of patients with abscess > 4 cm who underwent surgical treatment

	Type of surgery	Surgical approach	Clavien–Dindo ≥ 3	Readmission	Resurgery	Stoma reversal
HP	2	1 lpt, 1 conv	0	0	0	2
PA	23	10 lpt, 12 vls, 1 conv	0	1	0	–
PA + ileo	3	3 conv	0	0	0	3
Total	28	28	0	0	0	5

*HP* Hartmann’s procedure, *PA* colic resection and primary anastomosis, *PA* + *ileo* primary anastomosis with ileostomy, *lpt* laparotomy, *vls* laparoscopy, *conv* initial laparoscopy converted into open approach

No patient developed severe postoperative complications (Clavien–Dindo ≥ 3) and no patient required blood transfusions. Clavien–Dindo I events occurred in group 1, resolved by analgesic, antipyretic, antiemetic therapy alone. In group 2 the Clavien–Dindo II events were mainly related to ileus, in all cases resolved through the use of prokinetic drugs and/or placement of a nasogastric tube. The mean length of hospital stay was 8 days (± 4) for group 1 and 10 days (± 4) for group 2 (*p* = 0.033). A single patient required readmission for a phlegmon of the abdominal wall, successfully treated with i.v. antibiotic therapy. Stoma reversal was performed within 6 months from initial surgery in all cases and no surgical revision was necessary after primary surgery. None of the 6 patients with a stoma experienced complications during their subsequent hospitalization for stoma reversal surgery. The 3 patients, who experienced recurrence surgically treated with PA, did not develop post-operative complications.

With a minimum follow-up of 12 months (mean 36 months), 10 recurrences (16.7%) were observed in patients initially treated with antibiotic therapy only, none after resective surgery. Four recurrences occurred in group 1 (all patients previously treated with antibiotic therapy). Three of four patients required surgery and underwent PA, while in a single case conservative treatment was successfully retried. In group 2 we observed 6 recurrences, all in subgroup A. Three patients underwent PA, other 2 conservative treatments with i.v. antibiotic therapy, while in one case outpatient antibiotic therapy was successfully administered. Table 5 reports a comparison of the subgroups for recurrence rate.

**Table 5:** Comparison of disease recurrence rates according to treatment (antibiotic versus surgery) in each group

	Group 1 Abscess ≤ 4 cm (n = 43)			Group 2 Abscess > 4 cm (n = 57)		
	Atb (n = 31)	Atb + surgery (n = 12)	*p*	Atb (n = 29)	Atb + surgery (n = 28)	*p*
Recurrence, n (%)	4 (12.9)	0	0.563	6 (20.6)	0	**0.023**

Statistically significant *p*-value are displayed in bold

*Atb* antibiotic therapy

The analysis of the association between the type of antibiotic and conservative therapy failure highlighted that in both groups all the patients referred to surgery had been previously treated with i.v. amoxicillin-clavulanic acid (Table 6).

**Table 6:** Association between antibiotic regimen and failure of conservative therapy

	Group 1 Abscess ≤ 4 cm (n = 43)			Group 2 Abscess > 4 cm (n = 57)		
	Atb (n = 31)	Atb + surgery (n = 12)	*p*	Atb (n = 29)	Atb + surgery (n = 28)	*p*
Type of antibiotic, n (%)						
AC	19 (61.3)	12 (100)	**0.019**	15 (51.7)	28 (100)	** < 0.001**
PT	8 (25.8)	0	0.082	6 (20.7)	0	**0.023**
CM	4 (12.9)	0	0.563	8 (27.6)	0	**0.004**

Statistically significant *p*-values are displayed in bold

*Atb* antibiotic therapy, *AC* amoxicillin-clavulanic acid, *PT* piperacillin-tazobactam, *CM* ciprofloxacin-metronidazole

## Discussion and conclusions

DD is a frequent gastrointestinal disorder in Western Countries [11]. More than two hundred thousand people request hospital assistance every year for DD, impacting significantly on costs for the National Health System [12]. About 4% of these patients experience AD during their lifetime [13]. Their treatment is conservative in most cases, sometimes even handled as an outpatient [14, 15]. In complicated forms with purulent or faecal peritonitis, extensive bowel perforation or widespread abdominal collections, surgery is mandatory [7–9]. The preferred treatment for localized abdominal abscesses, found in about 25% of complicated ADs, is still debated [4]. Currently, recent guidelines recommend antibiotic therapy for abscesses up to 3 or 4 cm in diameter. When reachable, PD is recommended for those larger than 3 or 4 cm [7–9]. Nevertheless, little evidence is available to support this. Data regarding short and long-term outcomes of percutaneous treatments are heterogeneous and derive mainly from retrospective series. Many of these studies report no difference in success rate comparing PD vs antibiotic therapy [16, 17]. Even the assessment of the risk of recurrence is controversial. A systematic review has reported this to be overall higher in the antibiotic treatment group compared to PD, while for larger abscesses > 5 cm PD was associated with an increased risk of recurrence [18]. A recent large observational study reported that abscesses > 3 cm were associated with a higher short-term treatment failure compared to < 3 cm, while abscesses > 5 cm had an increased indication of surgery during short-term follow-up [19]. In addition, PD is not immune from complications, such as entero-cutaneous or entero-visceral fistulas, which sometimes require surgical treatment [20, 21]. Based on these controversial concerns, the practice of PD for DA never got into a routine at our Institution, although an extremely skilled interventional radiology department is available 24/7. Our policy is both due to the good results of resective surgery when antibiotics were not successful in controlling sepsis and for the uncertain management of each patient once the acute episode has been resolved.

In cases of complicated AD, Hartmann’s procedure is generally the most performed surgery, but a high rate of permanent colostomy is registered [22]. As an alternative, in selected cases laparoscopic lavage has been proposed [9]. The advantages and disadvantages of this technique have been extensively discussed in the literature, due to the conflicting results of the main trials SCANDIV, DILALA, LADIES and subsequent meta-analyses [23–26]. Due to the worse outcomes and the complications reported in some trials, guidelines suggest that laparoscopic lavage should not be considered as the first choice in diverticular peritonitis, being left as an option in selected cases [27–31].

Resection and PA with or without diverting stoma, initially performed as elective surgery, has been reconsidered in an emergency setting only in recent years. According to most recent guidelines, PA should represent the first therapeutic choice even in cases of severely complicated diverticulitis, such as in Hinchey III and IV disease, reserving HP and laparoscopic lavage only to high-risk patients with ASA scores ≥ 3 [32, 33]. Several randomized trials compared the outcomes of HP versus PA in this situation, finding no differences for in-hospital mortality or major complications rate (Clavien–Dindo IIIb–V). Diverting ileostomies, performed in some series in about one-third of the patients only, were more likely reversed than end colostomies [34, 35].

Similarly, even more we are convinced that for Hinchey Ib and IIa not responding to antibiotic therapy direct surgical resection with PA should be the preferred option. In a series of a hundred consecutive patients affected by DA, 40 not responding to antibiotic therapy underwent colonic resection. Short-term results showed a low incidence of complications and very few stomas required. The analysis of long-term data revealed that all stomas were reversed within a few months, with minimal impact on quality of life, together with a lower risk of recurrence within one year compared to conservative therapy.

In the past, surgery for AD was confined to an emergency setting due to the high perioperative morbidity and mortality. Nevertheless, these are more often dependent on clinical conditions, such as co-morbidities and sepsis, rather than the surgical technique itself. Currently, surgery is considered in patients with DAs Hinchey Ib and IIa after the failure of other treatments (antibiotics, percutaneous). We believe in these cases the role of surgery should be reconsidered as an effective one-way therapy with minimal sequelae. Hinchey Ib and IIa should be equated to symptomatic recurrent AD burdening quality of life. The present series shows that when peritonitis is confined and patients have a good performance status, PA experiences a very low rate of complications and need for temporary stomas with comparable results to elective delayed surgery. Indeed, percutaneous drainage may allow resolution of the acute episode and delay elective surgery in a referral centre. However, most peripheral centres are not equipped with interventional radiology even in daytime. Furthermore, percutaneous drainage would probably delay the timing of the operation, exposing the patient to a higher risk of complications, due both to the percutaneous treatment itself and to a prolonged infectious state.

So why not consider surgery as a *treatment of choice*, if, at the right timing, it can have good short- and long-term results? There is an increasing need for new contributions to this debate. Further data should contribute to evidence about the effectiveness of PA and PD for DA, supporting the definition of specific indications for both. Based on the aforementioned, an in-depth analysis of the role of different antibiotics regimens is also mandatory. Antibiotics should be effective against Grams—and anaerobes and oral use should be preferred over i.v. [9]. Although the choice of the molecule was depending on the operator’s preference, we observed that i.v. amoxicillin-clavulanic acid was significantly associated with failure of conservative therapy both in group 1 (*p* = 0.019) and in group 2 (*p* < 0.001). Furthermore, all patients who underwent surgery received amoxicillin-clavulanic acid initially. On the contrary, i.v. ciprofloxacin + metronidazole or piperacillin-tazobactam were associated with better infectious control compared to amoxicillin-clavulanic acid in group 2 (*p* = 0.023 and *p* = 0.004, respectively). When this would be confirmed in further studies, a more appropriate selection of antibiotics regimens may lead to a consistent reduction in the failure of sepsis control.

What also emerged from our work is that patients undergoing surgery were more likely to have a past history of acute uncomplicated diverticulitis. This is in contrast with previous studies [36], in which the history of diverticulitis is considered protective against the incidence of complicated diverticulitis. However, this result was not decisive in opting for surgery, which was instead adopted exclusively after the failure of antibiotic therapy.

Strengths of our study include a collection of a perspective database, sample homogeneity due to a reduced patient recruitment time and absence of missing data. Nonetheless, important limitations should be highlighted. Firstly, the monocentric retrospective nature of this work is the main source of bias. The sample consists of a small number of patients, although we believe it could be representative of the short recruitment time in our study. Finally, our study lacks antibiotic therapy management guidelines. Antibiotic regimen is left to the choice of each doctor at the time the patient enters the emergency room.

In conclusion, opting for resective surgery at the failure of antibiotic therapy in patients with DA, we observed a significant reduction in recurrence within 1 year, compared to antibiotic therapy, in patients with abscesses > 4 cm. Therefore, we suggest performing further studies comparing resective surgery with primary anastomosis to percutaneous drainage policies. 

## Data Availability

The datasets generated during and/or analysed during the current study are available from the corresponding author on reasonable request.
